# CCPE: cell cycle pseudotime estimation for single cell RNA-seq data

**DOI:** 10.1093/nar/gkab1236

**Published:** 2021-12-21

**Authors:** Jiajia Liu, Mengyuan Yang, Weiling Zhao, Xiaobo Zhou

**Affiliations:** College of Electronic and Information Engineering, Tongji University, Shanghai, Shanghai 201804, China; Center for Computational Systems Medicine, School of Biomedical Informatics, The University of Texas Health Science Center at Houston, Houston, TX 77030, USA; Center for Computational Systems Medicine, School of Biomedical Informatics, The University of Texas Health Science Center at Houston, Houston, TX 77030, USA; Center for Computational Systems Medicine, School of Biomedical Informatics, The University of Texas Health Science Center at Houston, Houston, TX 77030, USA; Center for Computational Systems Medicine, School of Biomedical Informatics, The University of Texas Health Science Center at Houston, Houston, TX 77030, USA; McGovern Medical School, The University of Texas Health Science Center at Houston, Houston, TX 77030, USA; School of Dentistry, The University of Texas Health Science Center at Houston, Houston, TX 77030, USA

## Abstract

Pseudotime analysis from scRNA-seq data enables to characterize the continuous progression of various biological processes, such as the cell cycle. Cell cycle plays an important role in cell fate decisions and differentiation and is often regarded as a confounder in scRNA-seq data analysis when analyzing the role of other factors. Therefore, accurate prediction of cell cycle pseudotime and identification of cell cycle stages are important steps for characterizing the development-related biological processes. Here, we develop CCPE, a novel cell cycle pseudotime estimation method to characterize cell cycle timing and identify cell cycle phases from scRNA-seq data. CCPE uses a discriminative helix to characterize the circular process of the cell cycle and estimates each cell's pseudotime along the cell cycle. We evaluated the performance of CCPE based on a variety of simulated and real scRNA-seq datasets. Our results indicate that CCPE is an effective method for cell cycle estimation and competitive in various applications compared with other existing methods. CCPE successfully identified cell cycle marker genes and is robust to dropout events in scRNA-seq data. Accurate prediction of the cell cycle using CCPE can also effectively facilitate the removal of cell cycle effects across cell types or conditions.

## INTRODUCTION

The rapid development of single-cell RNA-sequencing (scRNA-seq) technologies makes it possible to characterize cellular heterogeneity in gene expression at single-cell resolution (1–4). Accurate analysis of heterogeneous gene expression in single cells can help to better understand the specificity of any particular disease, thus discovering new genes as drug targets. There are many reasons for gene expression heterogeneity between cells, such as regulatory differences between cell types, cell cycle stage differences of the same type of cells, external microenvironment, etc. (5,6). Cell cycle, in particular, has been recognized as a key contributor of cell-to-cell gene expression variance (7,8). Numerous studies have demonstrated a tight association of cell cycle with cell fate decisions during development and tissue regeneration (9,10). During development/embryogenesis, embryonic stem cells/progenitor cells undergo self-renewal and lineage-specific differentiation programs to generate specific cell types. In adulthood, stem cells continue to differentiate and create fully differentiated progeny cells during tissue repair and normal cell renewal. Cell cycle plays an important regulatory role in cell fate decisions (9) and differentiation (10) of stem cells. As the main rate-limiting step of cell differentiation (10), cell cycle control is essential in ensuring generating cell diversity and maintaining the homeostasis of adult tissues. Cancer cells are derived from cancer stem cells/progenitor cells and can also be de-differentiated to re-enter the cell cycle and become cancer progenitor cells (11,12). Loss of cell cycle control can lead to uncontrolled tumor cell proliferation and growth (13). In addition to the significance in studies of tumorigenesis and development (14–16), the cell cycle is often regarded as a confounder in scRNA-seq data analysis when analyzing the role of other factors on transcriptional regulation. Removing confounder effects will improve the resolution of other biological processes (17). One of the strategies to remove cell cycle effects is removing cell cycle-related genes (18). Accurate prediction of cell cycles can help to identify cell cycle-related genes, thereby promoting the removal of cell cycle effects. Therefore, accurately identifying the cell cycle of individual cells is needed to fully understand a number of different biological problems and data analysis issues.

However, there are many challenges in predicting cell cycle using scRNA-seq data. Most of cell cycle-related informations are obtained through experimental methods, such as chemical induction (19), counterflow centrifugation elutriation (20) and DNA content (21). They have been used to detect the cell cycle phases of individual cells (22). These methods are time-consuming and laborious and not a quantitative measurement of cell cycle phase duration. Therefore, computational methods have been developed to determine cell cycle stages based on the transcription profile of the cells. The main computational methods currently used can be divided into two categories, knowledge-based and unsupervised. Knowledge-based methods, such as cyclone (8) and CellCycleScoring function in Seurat (23), use annotated cell cycle genes to predict the classification of each cell in G1, S or G2/M phase. Peco is another knowledge-based approach that uses the data generated from FUCCI fluorescence images and scRNA-seq to train the ‘naive Bayes’ predictor for predicting the progression position of each cell through the cell cycle process, which we called cell cycle pseudotime (6). Peco is specially designed for human induced pluripotent stem cells. reCAT requires cell cycle marker genes to calculate the Bayes-scores of each cell, and uses consensus traveling salesman problem (TSP) and hidden Markov model (HMM) to recover cell cycle pseudo time-series and stages (24). A common drawback of the knowledge-based methods is that their applications are limited to datasets with pre-annotated cell cycle genes and experimental cell cycle labels. To address this issue, several unsupervised methods have been proposed to predict cell cycle pseudotime, such as Cyclum (25) and CYCLOPS (26). Cyclum employs an autoencoder model that takes both non-linear and linear components in the hidden layer into account. The non-linear projection of gene expression profiles is trained to infer the pseudotime of cells in the circular process (25). CYCLOPS uses an autoencoder model with linear projection to project data onto a closed elliptical curve in low-dimensional space (26). However, CYCLOPS employs square root and division in the autoencoder model, which makes optimization more complicated. Using unsupervised methods to estimate the cell cycle not only has to consider how to model the circular process in the cell cycle, but also has to take the inherent characteristics of scRNA-seq data, sparsity and high dimensionality into account, which makes it difficult to use unsupervised methods to estimate the cell cycle.

In this study, we proposed a novel unsupervised method named CCPE to estimate cell cycle pseudotime of single cells from single-cell RNA-seq data. CCPE learns a discriminative helix to characterize the circular process and estimates pseudotime in the cell cycle. We assessed the performance of CCPE in estimating cell cycle pseudotime and cell stage assignment using both simulated and real scRNA-seq datasets. We also assessed the performance of CCPE in handling dropout events, analyzing smaller datasets with fewer genes or cells and removing the cell cycle effect from scRNA-seq data. We compared the performance of CCPE with the other methods, including cyclone, Seurat, Cyclum, CYCLOPS and reCAT.

## MATERIALS AND METHODS

### Datasets

We used both simulated datasets and real datasets (Supplementary Table S1) to evaluate the performance of CCPE.

#### Simulated scRNA-seq datasets

We simulated three datasets with different dropout rates (25.6%, 51.1% and 68.8%) using the simulation model in CIDR (27). Each simulated dataset contains three cell stages, representing G1, S, G2/M phases. One hundred fifty cells and 20,180 genes were generated for each simulated dataset by setting parameters *N* = 3, *k* = 50 in *scSimulator* function of CIDR package. Different dropout rates (25.6%, 51.1% and 68.8%) are achieved by setting the dropout level parameter *v* equal to 6.5, 9 and 12, respectively. Higher *v* means a higher level of dropouts.

#### mESCs Quartz-Seq dataset

The mouse embryonic stem cells (mESCs) were sequenced by Quartz-Seq technology, a reproducible and sensitive single cell RNA-seq method (21). This dataset has known cell cycle phases. Therefore, we used this dataset to evaluate the performance of different methods. Many other studies have used this dataset in cell-cycle analysis (8,24,28). The mESCs Quartz-Seq dataset has 35 mouse embryonic stem cells, including 20 cells in G1 phase, seven cells in S phase and eight cells in G2/M phase. mESCs Quartz-Seq dataset is available from Gene Expression Omnibus (GEO) with GEO Series ID GSE42268.

#### H1 hESCs scRNA-seq dataset

To compare the performance of CCPE and Cyclum on the data with different gene and cell sizes, especially the data with small number of genes and cells. We randomly subsampled the scRNA-seq data from human embryonic stem cells (GSE64016). This dataset consists of 247 cells and 19 084 genes. We selected seven gene sizes, ranging from 50 to 600 genes, and five cell sizes, ranging from 10 to 100 cells. Each data with a specific size was sampled 10 times for fair evaluation. Normalized expected counts were provided in this dataset and the cell cycle phases of 247 cells were identified using Fluorescent Ubiquitination-based Cell Cycle Indicator (FUCCI).

#### E-MTAB-2805 mESCs dataset

This scRNA-seq dataset of mouse embryonic stem cells were generated by Buettner *et al.* (28). The dataset was downloaded from https://www.ebi.ac.uk/arrayexpress/experiments/E-MTAB-2805/. The cells were stained with Hoechst and sorted using FACS for respective cell-cycle fractions (G1, S and G2/M phase). Two hundred eighty-eight mouse embryonic stem cells were sequenced using HighSeq 2000 sequencing system.

#### Nutlin-treated multiple cancer cell lines dataset

This dataset consists of two 10× single-cell RNA-seq data from nutlin-treated cells and control group. A mixed culture of 24 cell lines were treated with either dimethyl sulfoxide (DMSO) or nutlin. This dataset was downloaded from https://figshare.com/s/139f64b495dea9d88c70. Nutlin is known to elicit cell cycle arrest exclusively in cells expressing wild-type (WT) *TP53* (29). Thus, seven cell lines expressing WT *TP53* were used in CCPE to characterize the cell cycle effect of a cell cycle perturbation.

#### 416B cell line scRNA-seq dataset

416B cell dataset contains two 96-well plates of 416B cells (an immortalized mouse myeloid progenitor cell line) (30). It is processed using the Smart-seq2 protocol (31). The *CBFB-MYH11* oncogene was expressed in half of the cells and silent in the other half of cells (control). This dataset was downloaded from https://www.ebi.ac.uk/arrayexpress/experiments/E-MTAB-5522/.

### Normalization and pre-processing

For the mESCs Quartz-Seq data and E-MTAB-2805 data with FPKM and TPM expression levels, and other datasets with read counts for expression levels, we normalized the single cell RNA-seq datasets by taking log 2 transformation with a pseudo count 1 as

(1) $$\begin{equation*}{{\rm log}_2}\left( {{\rm FPKM} \left| { {\rm TPM} } \right|{\rm Counts} + 1} \right)\end{equation*}$$

Gene selection is recommended for the data pre-processing in CCPE. ‘dpFeature’ is a powerful and general approach for unsupervised feature selection to solve the sparsity problem in scRNA-seq data (32). ‘dpFeature’ excludes genes that are expressed in <5% of all the cells and selects the significantly differentially expressed genes for CCPE (32).

### Construction of CCPE model

#### Learning a helix in the reduced dimension

We suppose scRNA-seq data $X$ with $N$ cells and $D$ genes lies in the high dimension. In CCPE, we consider the linear projection $Z = f ( X ) = {W^T}\ X$ to infer the embedded expression profiles ${Z_{d \times N}}\ ( {d \ll D} )$ from $X$ and the reversed linear projection is $X\ = {f^{ - 1}}\ ( Z ) = WZ$, where $d$ is the reduced dimension and $W \in {R^{D \times d}}$ with ${W^T}W = I$. We construct a circular helix $\hat{Z} = f( {x,y,z} )$ in 3D dimension to get the best fit of Z, a circular helix of radius $a$ and slope $v/a$ (or pitch $2\pi v$) is described as follows for cell $i$

(2) $$\begin{eqnarray*} \widehat {{Z_i}} = {f_{Helix}} \left( {\widehat {{x_i}}, \widehat {{y_i}}, \widehat {{z_i}}} \right) = \left\{ \begin{array}{@{}*{1}{c}@{}} {\widehat {{x_i}} = v{\theta _i}}\\ {\widehat {{y_i}} = a\sin {\theta _i}}\\ {\widehat {{z_i}} = a\cos {\theta _i}} \end{array}\right. \end{eqnarray*}$$

Then we formulate the following object function to obtain the reduced dimension via learning a helix

(3) $$\begin{equation*}\mathop {\min }\limits_{W,Z,\hat{Z}} \mathop \sum \limits_{i = 1}^N \|{X_i} - W{Z_i}{\|^2} + \mathop \sum \limits_{i = 1}^N \|{Z_i} - \widehat {{Z_i}}{\|^2}\end{equation*}$$

$$\begin{eqnarray*} && s.t. \\ && \quad {W^T}W = I \\ && \quad \widehat {{Z_i}} = {f_{Helix}} \left( {\widehat {{x_i}},\ \widehat {{y_i}}, \widehat {{z_i}}} \right) = \left\{ \begin{array}{@{}*{1}{c}@{}} {\widehat {{x_i}} = v{\theta _i}}\\ {\widehat {{y_i}} = a\sin {\theta _i}}\\ {\widehat {{z_i}} = a\cos {\theta _i}} \end{array}\right. \\ && \quad a >0, v > 0 \end{eqnarray*}$$

where $X = [ {{X_1},{X_2}, \ldots ,{X_N}} ] \in {R^{D \times N}}$ is the scRNA-seq data, $W = [ {{W_1},{W_2}, \ldots ,{W_d}} ] \in {R^{D \times d}}$ is an orthogonal set of $d$ linear basis vectors ${W_l} \in {R^D}$, $Z = [ {{Z_1},{Z_2}, \ldots ,{Z_N}} ] \in {R^{d \times N}}$ is represented by the embedded expression profiles of $X$ in low-dimension ${R^d}$. $\hat{Z}$ contains fitted points of $Z$ on a circular helix with the same dimension as $Z$ and $\widehat {{x_i}}, \widehat {{y_i}}, \widehat {{z_i}}$ is the coordinates of cell $i$ projected on the circular helix.

Furthermore, cells in the same cell cycle phase should cluster together on the helix, so we consider the clustering objective into the optimization problem as below

(4) $$\begin{eqnarray*} && {\mathop {\min }\limits_{W,Z, \hat{Z}, Y} \mathop \sum \limits_{i = 1}^N {{\left\| {{X_i} - W{Z_i}} \right\|}^2} + \lambda \mathop \sum \limits_{i = 1}^N {{\left\| {{Z_i} - \widehat {{Z_i}}} \right\|}^2}} \nonumber \\ && \quad +\, \gamma \left[ {\sum\nolimits_{k = 1}^K {\sum\nolimits_{i = 1}^{{N_k}} {{r_{i,k}}{{\left\| {{Z_i} - {Y_k}} \right\|}^2} + \sigma {\rm{\Omega }}\left( R \right)} } } \right] \nonumber \\ && s.\ t.\ \ {W^T}W = I, \widehat {{Z_i}} = {f_{Helix}} , \mathop \sum \limits_{k = 1}^K \ {r_{i,k}} = 1, {r_{i,k}} \ge 0, \forall i, \forall k \nonumber \\ \end{eqnarray*}$$

where ${N_k}$ is the number of cells in cluster $k$. ${r_{i,k}}$ is the weight of soft clustering based on the assumption of $K$ clusters. ${Y_k}$ represents the centroid of cluster $k$. ${\rm{\Omega }}( R )$ is the negative entropy regularization and $\sigma >0$ is the regularization parameter for ${\rm{\Omega }}( R )$. $\lambda >0,\ \gamma > 0$ are parameters that indicate the importance of each component of the objective function. The solution of ${r_{i,k}}$ in terms of $\mathop \sum \limits_{k\ = \ 1}^K \ {r_{i,k}} = \ 1$ and formula of ${\rm{\Omega }}( R )$ are described in (33) as the following

(5) $$\begin{equation*}{r_{i,k}} = \exp \left( { - \frac{{\|{Z_i} - {Y_k}{\|^2}}}{\sigma }} \right) \bigg/\mathop \sum \limits_{k = 1}^K \exp \left( { - \frac{{\|{Z_i} - {Y_k}{\|^2}}}{\sigma }} \right)\end{equation*}$$

(6) $$\begin{equation*}{\rm{\Omega }}\left( R \right) = \mathop \sum \limits_{i = 1}^N \mathop \sum \limits_{k = 1}^K {r_{i,k}}log{r_{i,k}} \end{equation*}$$

#### Optimization of CCPE’s objective function

We optimize the object function (4) using alternating structure optimization, which has been successfully applied to several optimization problems (34). We divide the parameters to be optimized into two parts $\{ {W,\ Z,\ Y} \}$and $\{ {\hat{Z}} \}$ and solve one group by fixing the other group alternatively until convergence.

Firstly, we optimize $\{ {W,\ Z,\ Y} \}$ by fixing $\{ {\hat{Z}} \}$. Given a known helix, we can see $\hat{Z}$ as a constant matrix $C \in {R^{d \times N}}$. After simple matrix manipulation (Supplementary Text S1), function (4) with respect to $\{ {W,\ Z,\ Y} \}$ can be rewritten as the following optimization problem

(7) $$\begin{eqnarray*} && \mathop {\min }\limits_{W,Z,Y} tr\left[ X{X^T} - 2{W^T}X{Z^T} + \left( {1 + \lambda + \gamma } \right)Z{Z^T} \right. \nonumber \\ && \quad \left. - 2\lambda C{Z^T} + \lambda C{C^T} - 2\gamma {R^T}{Z^T}Y + \gamma Y{\rm{\Gamma }}{Y^T} \right] \end{eqnarray*}$$

where ${\rm{\Gamma }} = diag( {{1^T}R} )$ and $R$ is the weight matrix of soft clustering. Set $L$ equals formula (7) and the first derivative of $L$ with respect to $Y$ to zero

(8) $$\begin{equation*}\frac{{\partial L}}{{\partial Y}} = - 2\gamma ZR + 2\gamma Y{\rm{\Gamma }} = 0\end{equation*}$$

Then we get the optimization of $Y$ as

(9) $$\begin{equation*}Y = ZR{{\rm{\Gamma }}^{ - 1}}\end{equation*}$$

Substituting $Y$ into $L$ and set the first derivative of $L$ with respect to $Z$ to zero, we can get the optimization of $Z$ as

(10) $$\begin{eqnarray*} Z &=& \left( {{W^T}X + \lambda C} \right) {{[\left( {1 + \lambda + \gamma } \right)I - \gamma R{{\rm{\Gamma }}^{ - 1}}{R^T}]}^{ - 1}} \nonumber \\ &=& \left( {{W^T}X + \lambda C} \right) Q \end{eqnarray*}$$

where $Q = {[( {1 + \lambda + \gamma } )I - \gamma R{{\rm{\Gamma }}^{ - 1}}{R^T}]^{ - 1}}$ and the inverse of $[ {( {1 + \lambda + \gamma } )I - \gamma R{{\rm{\Gamma }}^{ - 1}}{R^T}} ]$ exists (Supplementary Text S2). Similarly, substituting $Z$ into $L$, the objective function becomes the following optimization problem

(11) $$\begin{equation*}L = \mathop {\max }\limits_W tr\left( {CQ{X^T}W} \right): {W^T}W = I \end{equation*}$$

This is the constrained quadratic problem (Supplementary Text S3) which has the closed-form solution (35) of $W$ as follows

(12) $$\begin{equation*}W = V{I_{D \times d}}{U^T}\end{equation*}$$

where $V$ is an $D \times D$ unitary matrix, $U$ is an $d \times d$ unitary matrix, and $U\Sigma {V^T}$ is the singular value decomposition of matrix $CQ{X^T}$, $\Sigma$ is an $d \times D$ rectangular diagonal matrix with non-negative real numbers on the diagonal.

Secondly, given $W, Z$ and $Y$, we can obtain $\hat{Z}$ easily by solving the following curve fitting problem

(13) $$\begin{equation*}\mathop {\min }\limits_{a,v,\theta } \mathop \sum \limits_{i = 1}^N \|{Z_i} - \widehat {{Z_i}}{\|^2}: \widehat {{Z_i}} = {f_{Helix}} \end{equation*}$$

Overall, the optimization process of the problem (4) is given in Algorithm 1 and the dimensions of all the matrices used in CCPE were provided in Supplementary Table S2.

**Table:** 

**Algorithm 1**	
1.	**Input:** scRNA-seq data $X$, parameters $\lambda ,\ \sigma$ and $\gamma$, number of
	clusters $K$.
2.	Initialize $Z$ and $Y$
3.	**Repeat**
4.	Obtain $\hat{Z}$ by solving (13) via Helix fitting
5.	Compute $R$ with each element as (5)
6.	$\Gamma = diag( {{1^T}R} )$
7.	$Q = {[( {1 + \lambda + \gamma } )I - \gamma R{\Gamma ^{ - 1}}{R^T}]^{ - 1}}$
8.	$A = \hat{Z}\ Q{X^T}$
9.	Perform SVD on $A$ such that $A = U\Sigma {V^T}$
10.	$W = V{I_{D \times d}}{U^T}$
11.	$Z = ( {{W^T}X + \lambda \hat{Z}} ) Q$
12.	$Y = ZR{\Gamma ^{ - 1}}$
13.	**Until** Convergence

#### Strategy for setting weighting parameters in CCPE

There are three parameters $\lambda$, $\sigma$ and $\gamma$ in the objective function and they represent the weights of multi tasks in CCPE. $\lambda$ regulates the coverage of Helix, $\gamma$ regulates the performance of clustering and $\sigma$ represents the weight of negative entropy regularization. We used both scRNA-seq datasets with ground truth and without ground truth in CCPE. For the datasets with ground truth, we firstly set the initial values of the parameters empirically, then fix two parameters and change the value of another parameter until the model gets its best performance (36). Taking E-MTAB-2805 mESCs dataset as example, the objective function values of CCPE converge as the number of iterations increases (Supplementary Figure S1). We used the accuracy as a clustering evaluation criteria to assess the performance of CCPE with respect to parameters $\{ {\lambda , \gamma , \sigma } \}$ and CCPE achieved the best performance when $\lambda = 70$, $\gamma = 140$ and $\sigma = 0.01$ (Supplementary Figure S2). The same strategy was also used on the mESCs Quartz-Seq dataset and H1 hESCs scRNA-seq dataset. CCPE has a good performance on mESCs Quartz-Seq dataset and H1 hESCs scRNA-seq dataset when setting the values of $\{ {\lambda , \gamma , \sigma } \}$ to {50, 50, 0.001}. For the datasets without ground truth, we artificially set parameters $\{ {\lambda , \gamma , \sigma } \}$ to {50, 50, 0.001} in order to avoid tuning too many parameters.

## RESULTS

### Overview of CCPE approach

**Figure 1.**
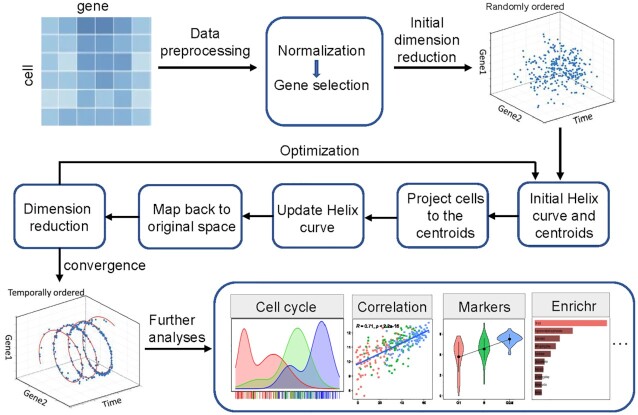
Overview of CCPE approach. After normalization and pre-processing of the data, CCPE learns the discriminative helix by iteratively optimization between the original and reduced dimension until convergence. After optimization, a 3D-helix with two-gene dimensions is used to represent circular information of cell cycle phases and one dimension to represent pseudotime of cells along the cell cycle. Several downstream analyses and applications of CCPE are used to assess its performance.

Single-cell RNA sequencing (scRNA-seq) data is a cell-specific gene expression matrix with high dimensionality and sparsity. Traditional clustering methods have low efficiency for computing high-dimensional and sparse matrices. Therefore, it is necessary to introduce dimension reduction in the model. We develop CCPE, a novel cell cycle pseudotime estimation method to characterize cell cycle timing from single-cell RNA-seq data. CCPE maps high-dimensional scRNA-seq data onto a helix in three-dimensional space, where 2D space is used to capture the cycle information in scRNA-seq data, and one dimension to predict the chronological orders of cells along the cell cycle, which we called cell cycle pseudotime (Supplementary Figure S3). ScRNA-seq data is repeatedly transformed from high dimension to low dimension and then mapped back to high dimension. At the same time, CCPE iteratively optimizes the discriminative dimensionality reduction via learning a helix until convergence (Figure 1). CCPE is applied to several analyses and applications to demonstrate its ability to accurately estimate the cell cycle pseudotime and stages.

### Estimation of cell cycle pseudotime

**Figure 2.**
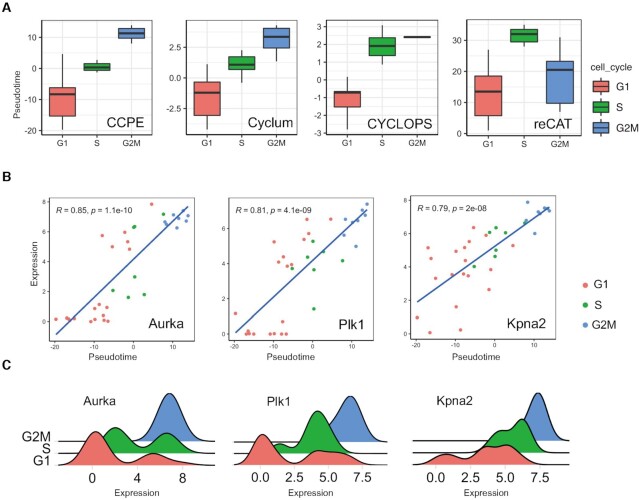
Cell cycle pseudotime analysis of mESCs Quartz-Seq data. (**A**) Boxplots demonstrated the distribution of cell cycle pseudotimes inferred by CCPE, Cylum, CYCLOPS and reCAT, respectively. Each box was colored by the corresponding cell cycle phases and outliers were ignored in the figure. (**B**) The expressions of three cell cycle marker genes are highly correlated with the cell cycle pseudotime estimated by CCPE. The correlation coefficients and p-value are shown on the top left of each figure. (**C**) shows density plots of the expressions of three G2/M-phase marker genes.

As we mentioned in the introduction, few computational tools have been developed so far to be used for the estimation of cell cycle pseudotime for scRNA-seq data, including Cyclum, CYCLOPS and reCAT (24–26). To test the performance of CCPE in predicting the cell cycle pseudotime, we compare the performance of CCPE with Cyclum, CYCLOPS and reCAT based on scRNA-seq data of mouse embryonic stem cells (mESCs) sequenced by Quartz-Seq technology (21). This dataset has known cell cycle phases that can be used as the golden standard to evaluate the performance of different models. Figure 2A illustrates the distribution of cell cycle pseudotime estimated by each method. Both CCPE and Cyclum can maintain the correct cell cycle order from G1 to S, and then to G2/M. Both of CYCLOPS and reCAT can distinguish G1 and S phases well but do not characterize G2/M phase in the right order after S phase. Compared with Cyclum, CCPE shows a better performance in separating S and G2/M phases. To test whether the pseudotime estimated by CCPE is significantly differential for G1, S and G2/M phases, we performed Analysis of Variances (ANOVA) (37) on three groups (G1, S and G2/M phases). The ANOVA result (Supplementary Table S3) shows the variance of the three groups differed significantly and the p-value of the ANOVA result is 1.24E–09, which confirms that the pseudotime estimated by CCPE is significantly differential for G1, S and G2/M phases.

We calculated the Pearson correlation of the gene expression and cell cycle pseudotime inferred by CCPE. Aurora kinase A (*Aurka*), polo-like kinase 1 (*Plk1*) and karyopherin alpha 2 (*Kpna2*) have the highest correlation with cell cycle pseudotime. The correlation coefficients of *Aurka*, *Plk1* and *Kpna2* genes are 0.85, 0.81 and 0.79, respectively (Figure 2B). *Aurka* is known as a key cell-cycle regulator, whose levels of mRNA and protein are low in G1 and S and increase sharply during G2/M phase (38). *Plk1* has a crucial role in the regulation of mitotic checkpoints and is active in the late G2 phase (39). Knocking-down *Kpna2* has been shown to inhibit cell proliferation by inducing cell cycle arrest in G2/M phase (40). We found that the most highly correlated genes with cell cycle pseudotime are G2/M-phase marker genes (Supplementary Figure S4), which are all highly expressed in G2/M phase (Figure 2C, Supplementary Figure S5).

### Assignment of cell cycle stages

**Figure 3.**
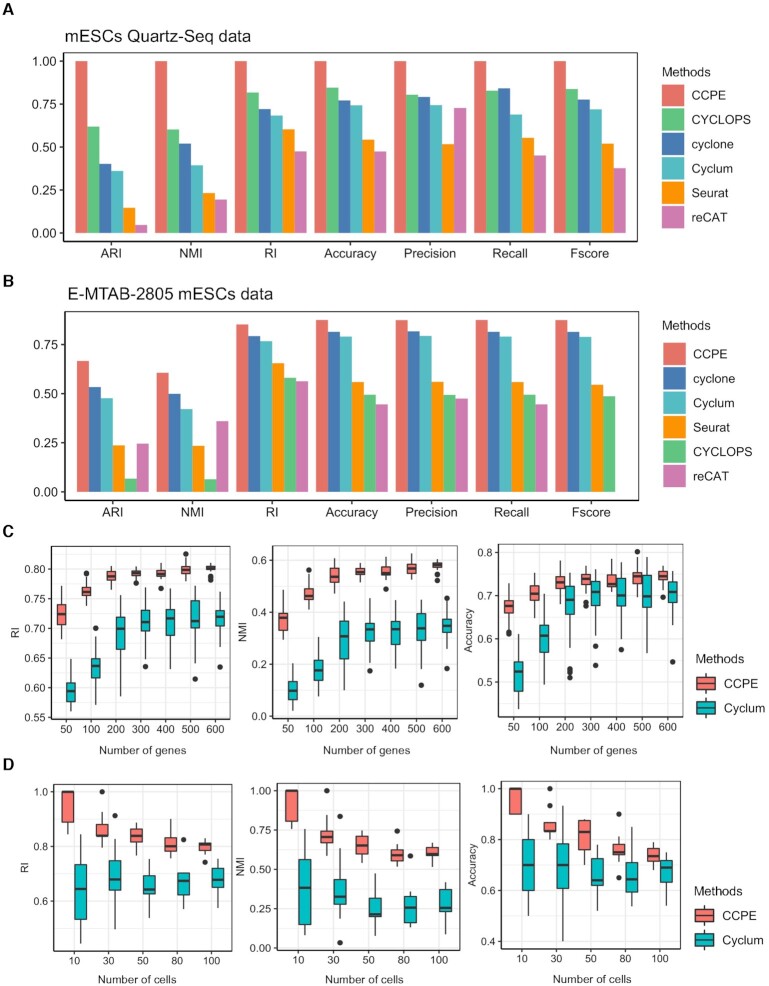
Cell-cycle stage inference from real datasets. (**A**) shows seven multiclass classification metrics to evaluate the cell cycle classification accuracy of CCPE, cyclone, Seurat, reCAT, Cyclum and CYCLOPS for mESCs Quartz-Seq data. (**B**) shows seven multiclass classification metrics to evaluate the cell cycle classification accuracy of CCPE, cyclone, Seurat, reCAT, Cyclum and CYCLOPS for E-MTAB-2805 mESCs data. Details of all the clustering measurements are provided in Supplementary Text S4. (**C**) Boxplots of RI, NMI and Accuracy values indicate the performance of CCPE and Cyclum on the subsampled datasets with smaller number of genes. (**D**) Boxplots of RI, NMI and Accuracy show the cell cycle classification accuracy of CCPE and Cyclum on the subsampled datasets with different numbers of cells.

We compared the competence in assigning cells into the correct cell cycle stages of CCPE with others models. To do so, we took advantage of a Gaussian mixture model with three components to transform the continuous pseudotime generated by CCPE, Cyclum and CYCLOPS into discrete cell cycle stages. In addition to Cyclum and CYCLOPS, we also compared CCPE with cyclone, Seurat and reCAT using both mESCs Quartz-Seq and E-MTAB-2805 mESCs datasets. Seven classification metrics were used to evaluate the models’ performance. Precision, Recall and Fscore represent Macro-Precision, Macro- Recall and Macro-Fscore for multiclass classification evaluation, separately. The details of clustering metrics are described in Supplementary Text S4. Due to the randomness in the machine learning models, each method was evaluated ten times on each dataset and the average value of each clustering metric was recorded (Supplementary Table S4, Supplementary Table S5). CCPE has an excellent performance in analyzing mESCs Quartz-Seq dataset, with highest values of clustering metrics among all methods (Figure 3A). CCPE also performs very well in analyzing the E-MTAB-2805 mESCs dataset, ranking first in all of individual metrics (Figure 3B). The performance of the knowledge-based method cyclone is second only to CCPE and reCAT can not calculate Macro-Fscore for E-MTAB-2805 mESCs dataset. The overall performance of Cyclum is better than Seurat, CYCLOPS and reCAT (Supplementary Table S5). Our results demonstrate the excellent performance of CCPE in predicting the cell cycle stages.

### Robustness of CCPE in analyzing small size of scRNA-seq data

To evaluate the performance of CCPE on the data with different numbers of genes and cells, especially the data with a small number of genes and cells, we randomly subsampled the scRNA-seq data from the human embryonic stem cell dataset, which consists of 247 cells and 19 084 genes. We selected seven sub-datasets with various number of genes, ranging from 50 to 600 genes, and five sub-datasets with various cell numbers, ranging from 10 to 100 cells. We found that the median of all the clustering metrics of both CCPE and Cyclum gradually increased with the number of genes (Figure 3C, Supplementary Figure S6). CCPE consistently outperformed Cyclum in terms of seven clustering metrics. In other words, CCPE can predict cell cycle stages more accurately based on a smaller number of genes than Cyclum. CCPE also has better performance on a smaller number of cells compared with Cyclum. The performance of CCPE gradually declines as the number of cells increases and finally stabilizes (Figure 3D, Supplementary Figure S7). The median value of Cyclum oscillates within a certain range (between 0.65 and 0.68 in RI, between 0.23 and 0.38 in NMI and between 0.63 and 0.7 in Accuracy), but lower than CCPE. Our analysis indicates that CCPE is more robust and has a higher prediction accuracy for the datasets with a smaller number of genes or cells.

### Differential gene expression analysis based on inferred cell cycle phases

**Figure 4.**
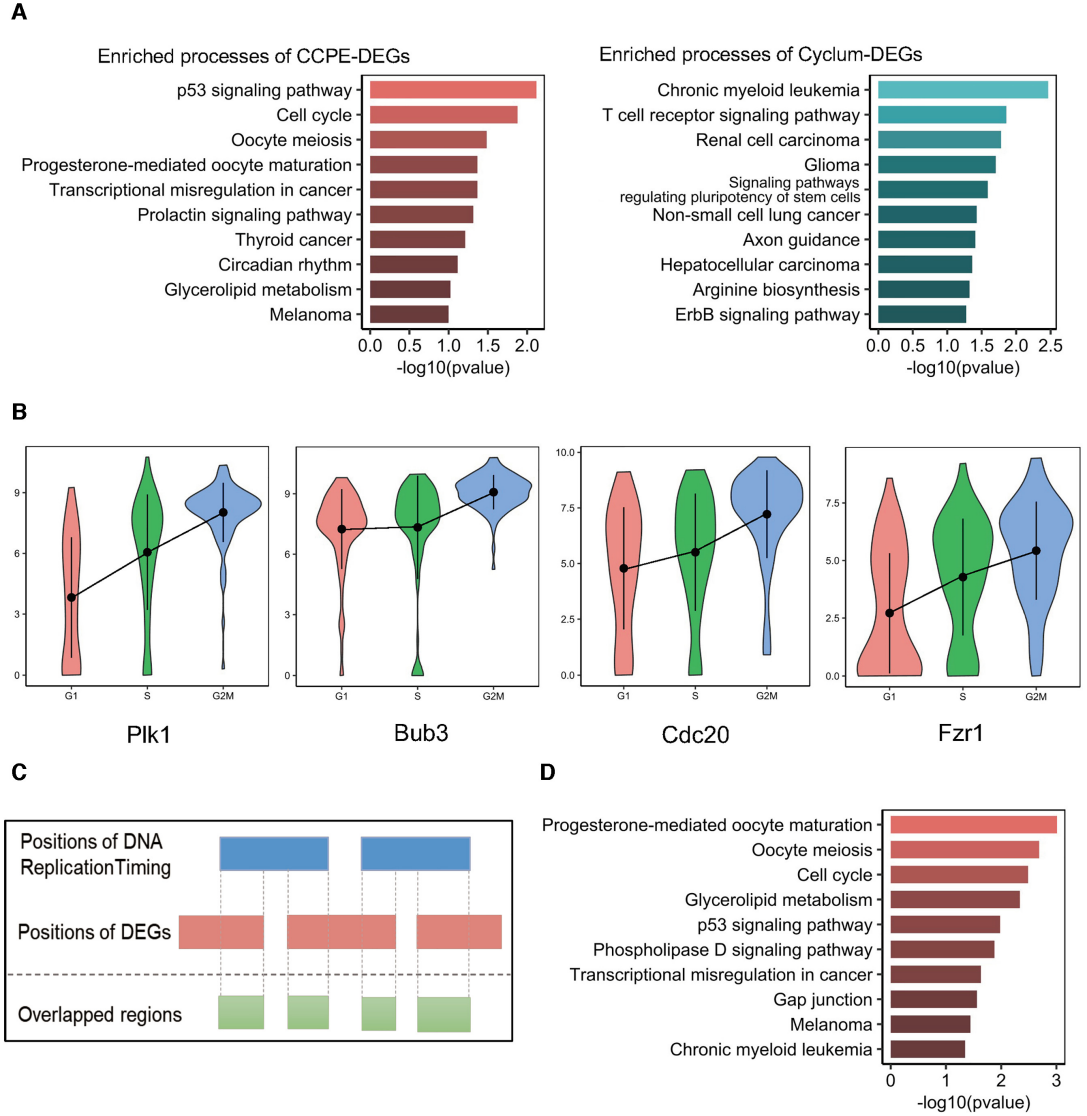
Differentially expressed gene analysis on E-MTAB-2805 mESCs data. (**A**) Top ten enriched biological progresses associated with DEGs identified by CCPE-inferred and Cyclum-inferred cell cycle stages. (**B**) The violin plots show the expression of G2/M phase marker genes Plk1, Bub3, Cdc20 and Fzr1. (**C**) Schematic representation of the intersection between DEGs and DNA replication timing events. (**D**) Top ten enriched biological progresses of the 682s DEGs overlapped with DNA replication timing events.

Differential gene expression analysis of inferred cell cycle phases can identify gene expression variability between different cell cycle phases. We use DESeq2 (41) implemented in R/Bioconductor to detect differentially expressed genes (DEGs) from CCPE-inferred and Cyclum-inferred cell cycle stages (*P*.adjusted ≤ 0.05 and |log_2_FC| ≥ 1) for E-MTAB-2805 mESCs data. Gene set enrichment analysis (42) shows that the DEGs identified by CCPE are mainly involved in the cell cycle pathways and enriched in the biological cell cycle-related processes, including p53 signaling pathway, progesterone-mediated oocyte maturation and circadian rhythm. The DGEs identified by Cyclum have little relationship with the cell cycle (Figure 4A). Figure 4B shows the expression of four G2/M phase marker genes *Plk1*, *Bub3*, *Cdc20* and *Fzr1*, which are enriched in the cell cycle pathway.

Studies indicated that the DNA replication timing program of the cell is highly organized and defined as the temporal sequence of locus replication events during the S phase of the cell cycle (43,44). During the S-phase of each cell cycle, all of the DNA in a cell is duplicated in order to provide one copy to each of the daughter cells after the next cell division. DNA replication timing is the temporal order of DNA replication of all the segments in the genome. To further investigate whether these differentially expressed genes are associated with the cell cycle, we intersected the positions of 1577 DEGs identified by CCPE on the chromosomes with DNA replication timing events of the human genome using *intersect* function in BEDTools (45) (Figure 4C). We found that 682 out of 1557 DEGs are overlapped with features in DNA replication timing events (Supplementary Figure S8). Enrichment analysis of the overlapped genes shows the enriched terms are mainly associated with the regulation of the cell cycle processes (Figure 4D). Our results further confirms the accuracy of CCPE in predicting cell cycle stages and identifying cell cycle related genes.

### Robustness of CCPE in dealing with dropout events of scRNA-seq data

**Figure 5.**
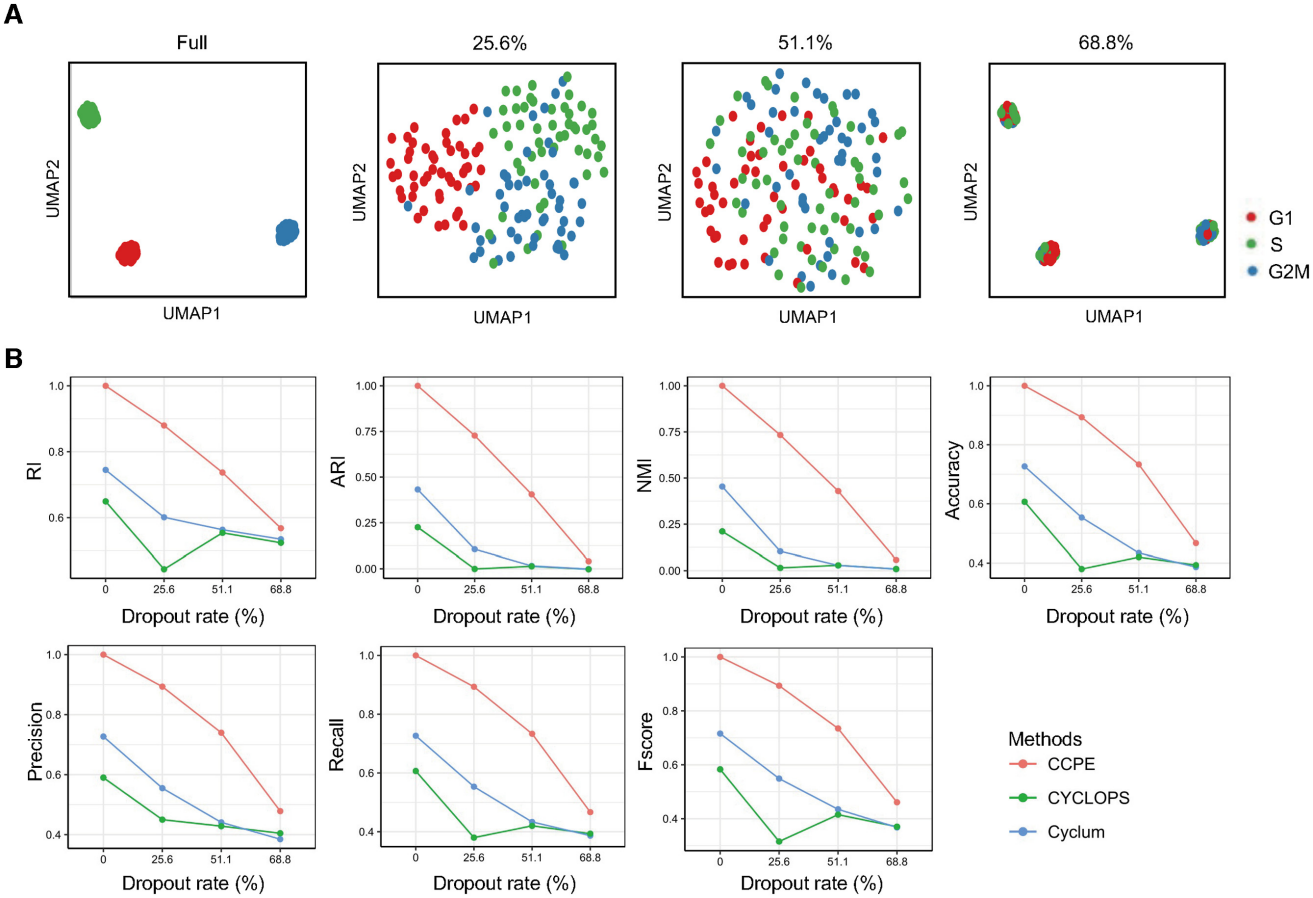
Robustness of CCPE in dealing with dropout events in simulated datasets. (**A**) UMAP plots for data with 0%, 25.6%, 51.1% and 68.8% dropout rate. Full represents the data without dropout events. Cells were colored by CCPE-inferred cell cycle stages. (**B**) The impact of dropout rate on the performance of CCPE, Cyclum and CYCLOPS evaluated by seven multiclass classification metrics using simulated datasets. The methods are marked with different colors.

scRNA-seq data always suffers from many sources of technical noises, leading to excess false zero values, which are termed as dropout events (46). The tools developed for analyzing scRNA-seq data should take their ability to handle dropout events into account. We used three simulated datasets with different dropout rates (25.6%, 51.1% and 68.8%) generated by *scSimulator* function in CIDR (27) to evaluate the robustness of CCPE in dealing with dropout events. Figure 5A shows the UMAP visualization of simulated cells at different CCPE-inferred cell cycle stages. As the dropout rate increases, the performance of CCPE on separating three cell cycle clusters gradually decreases. When the dropout rates are 51.1% and 68.8%, it is difficult for CCPE to distinguish the three cell cycle phases (Figure 5A). As we all know, the higher the dropout rate, the more gene expression are lost. We compared the impact of the dropout rate on the performance of CCPE, Cyclum and CYCLOPS. By calculating clustering evaluation metrics (Figure 5B), we can see that when the dropout rate is less than 51.1%, CCPE performs significantly better than Cyclum and CYCLOPS. When the dropout rate increases to 68.8%, CCPE, Cyclum and CYCLOPS all performed poorly in estimating cell cycle phases. The values of clustering evaluation metrics of CCPE are still higher than Cyclum and CYCLOPS. Thus, our analysis indicates that CCPE is more robust to dropout events than Cyclum and CYCLOPS.

### Detection of G1 arrest in Nutlin-treated cancer cell lines using CCPE

**Figure 6.**
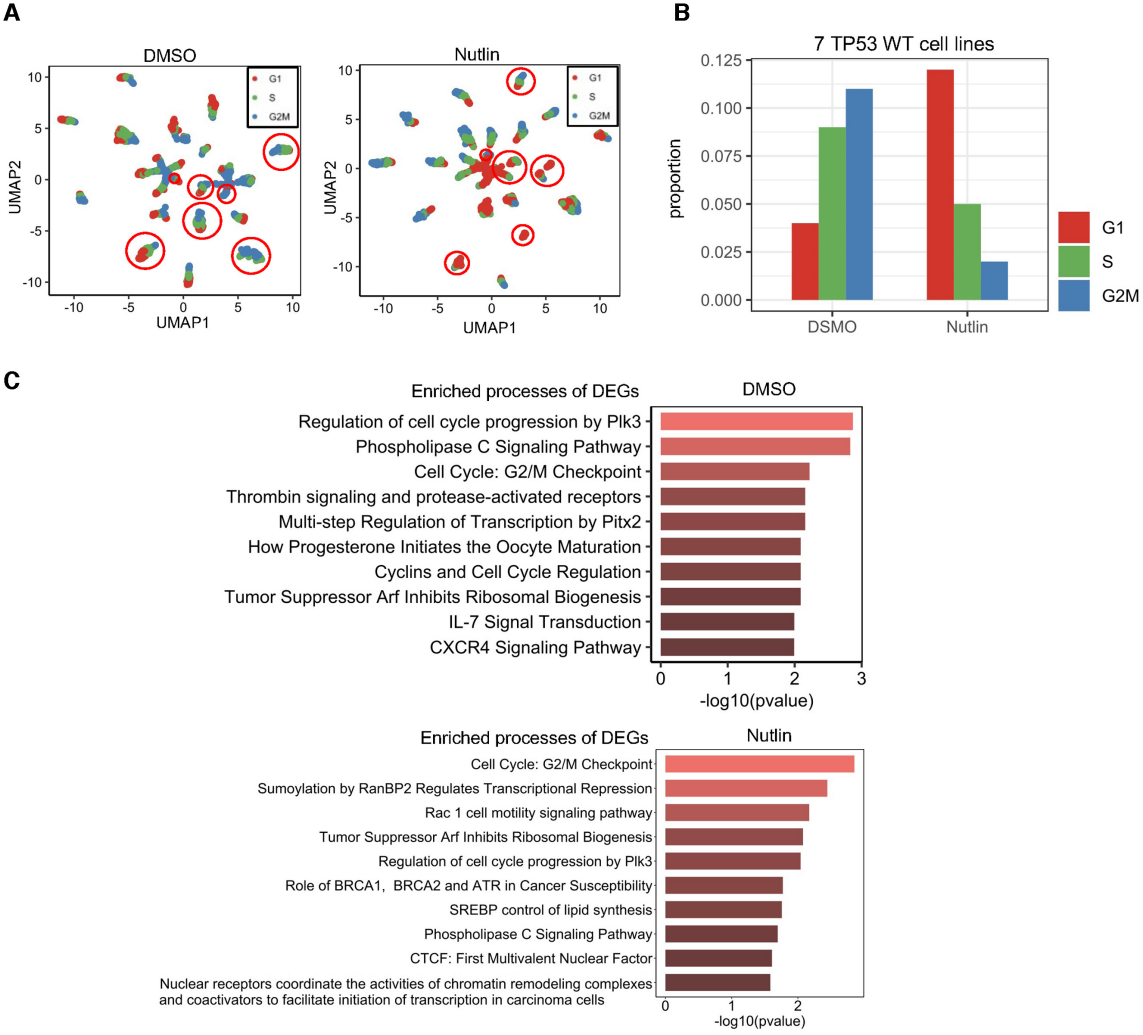
Validation of CCPE prediction accuracy using scRNA-seq datasets from cells treated with cell cycle disruptors. (**A**) UMAP plots for 24 cancer cell lines treated with DMSO and nutlin, separately. Cells were colored by CCPE-inferred cell cycle stages and cells in red circle are *TP53* WT cells. (**B**) The number of cells in CCPE-inferred cell cycle stages as a proportion of the total number of cells in DMSO- and nutlin-treated groups. (**C**) Top ten biological processes of DEGs identified from CCPE-inferred cell cycle stages.

To further assess the performance of CCPE, we applied CCPE to the cancer cell datasets with or without treatment with nutlin. Nutlin is a MDM2-p53 inhibitor (47) and can induce cell cycle arrest (48). One dataset was from the cells treated with vehicle DMSO and another one is from the same cells treated with nutlin (29). The cells used in culture were a cancer cell mixture with seven *TP53* WT cell lines and seventeen *TP53* mut cell lines. As shown in Figure 6A, *TP53* WT cells were in red circle and cells were colored by CCPE-infered cell cycle stages. Compared with the cells in the control group treated with DMSO, CCPE successfully detected an increase in the number of *TP53* WT cells in the G1 phase treated by Nutlin. (Figure 6A, Supplementary Figure S9). We screened out the data of the seven *TP53* WT cell lines and calculated the cell number ratio in each cell cycle phase. We found a significant increase of G1-phase cells, which confirmed that Nutlin can elicit a pronounced G1 arrest in *TP53* WT cells compared with the untreated control (Figure 6B). We also applied Deseq2 to identify the DEGs associated with CCPE-inferred cell cycle stages. It is obvious that some of the top 10 enriched pathways of these DEGs are associated with cell cycle, such as regulation of cell cycle progression and cell cycle G2/M checkpoint (Figure 6C). The enrichment analysis of DEGs further illustrates the accuracy of CCPE in estimating cell cycle stages and the reliability of CCPE to successfully detect G1 arrest in nutlin-treated *TP53* WT cells.

### Cell cycle effect removal from scRNA-seq data

Cells in different cycle stages may have quite different expression profiles, which can obscure the differences in expression between cell types and affect cell type identification and functional analysis of scRNA-Seq data (49). Therefore, it is important to remove cell cycle effect before conducting further analysis of scRNA-seq data. We use the murine multipotent myeloid progenitor cell line 416B dataset (30) to assess the performance of CCPE in removing cell cycle effect. We compute the percentage of variance explained by the CCPE-inferred cell cycle stages in the expression profile for each gene. Genes with high percentages are regarded as cell cycle-related genes and are removed from the dataset (18). We found that there is a small effect caused by cell cycle in the 416B dataset (Supplementary Figure S10). This dataset was generated from 416B cells with or without the expression of *CBFB-MYH11* oncogene. Therefore, two phenotypes exist in the dataset. After removing cell cycle effect using four methods, CCPE, Cyclum, Seurat and ccRemover, CCPE can separate two phenotypes correctly and the variation between two phenotypes was more pronounced compared with raw data (Supplementary Figure S11). Cyclum and Seurat can divide cells into two groups, but these two groups do not correspond to the expected phenotypes. CcRemover unables to distinguish between the two phenotypes (Supplementary Figure S10).

## DISCUSSION

Pseudotime analyses of single-cell RNA-seq data have been increasingly used to determine the latent pattern of dynamic processes experienced by cells, such as the cell cycle (50). We defined cell cycle pseudotime to describe the progression through the entire cell cycle process. Clustering is a common step to group cells into different cell cycle stages, learning gene expression patterns within different subgroups (51). It is widely used in single-cell transcriptomics workflows. However, cell cycle is a dynamic process that gene expression varies between cells not subgroups. Cell cycle pseudotime analysis attempts to characterize such differences by projecting cells along a continuous process rather than dividing cells into discrete clusters (6). In this study, we developed a novel cell cycle pseudotime estimation method named CCPE to accurately characterize cell cycle timing for single-cell RNA-seq data. CCPE learns a discriminative helix with two dimensions to characterize circular process in cell cycle and one dimension to symbolize the pseudotime of cells along the cell cycle process. This is a kind of task in manifold learning, a strategy to learn the intrinsic structure of complex and high-dimensional data. We used alternating structure optimization to fit the best helix from scRNA-seq data. The parameters were optimized in the iterative transformation of high and low dimensional spaces. Discriminative information of cells in the same cell cycle phase was taken into consideration during the optimization process. Although CCPE is designed to predict cell cycle pseudotime, it can convert the pseudotime into discrete cell cycle stages through a Gaussian mixture model. The Gaussian mixture model is defined as a linear combination of multiple Gaussian distributions, it is a common clustering method based on the estimation of the probability density distribution of the sample (52). In the Gaussian mixture model, we used three Gaussian distributions to represent G1, S and G2/M phases.

Gene selection is recommended during data pre-processing of CCPE. Since single cell RNA-seq data suffers from many sources of technical noise (53). Some cell cycle estimation methods only use cell cycle genes, such as cyclone, Seurat and reCAT. Cyclone applied thousands of cell cycle gene pairs to determine the cell cycle phases of cells (8). While in Seurat, only a small number of S phase marker genes (43) and G2M phase marker genes (54) are used to identify the cell cycle stages (23). The semi-supervised algorithm, reCAT, used 378 cell cycle genes listed in Cyclebase3 (54) to get gene expression matrix, while other genes were excluded based on the risk of adding noise to the model (24). Based on their performance on real scRNA-seq datasets, it is difficult to figure out how many cell cycle genes are sufficient to predict the cell cycle accurately. On the other hand, there are some genes that are influenced by cell heterogeneity and partially contribute to the cell cycle. If these genes were completely ignored, then additional noise would be introduced to the cell cycle prediction. Therefore, we recommended using a sophisticated approach called dpFeature to select differentially expressed genes during pre-processing of CCPE. dpFeature discovers the important ordering genes from the data, rather than relying on cell cycle marker genes from the literature (32).

We assessed the performance of CCPE in estimating cell cycle pseudotime and various applications using both simulated and real scRNA-seq datasets. Even though CCPE is an unsupervised algorithm, we compared it with both knowledge-based and other unsupervised algorithms, including cyclone, Seurat, Cyclum, CYCLOPS and reCAT. Peco is not included in the comparison since fluorescence imaging is required with scRNA-seq to measure cell cycle phases. The mESCs Quartz-Seq dataset is widely used in various cell cycle studies (8,24,28). We compared the performance of CCPE with several algorithms in characterizing the cell cycle pseudotime using mESCs Quartz-Seq dataset. CCPE not only captured the right order of three cell cycle phases, but also separated them very well as expected. Additionally, correlation analysis shows the genes highly correlated with CCPE-inferred cell cycle pseudotime are G2/M phase marker genes. Gaussian mixture model in CCPE was applied to estimate discrete cell cycle states. We calculated seven multiclass classification metrics on real datasets and our results indicated that CCPE had an outstanding performance compared with cyclone, Seurat, Cyclum, CYCLOPS and reCAT. We also tested the stability of CCPE in predicting cell cycle stages when the number of cells and genes in the dataset is small. Enrichment analysis showed that the DEGs identified by CCPE-inferred cell cycle stages had more connection to the biological processes related to cell cycle pathways. To evaluate the performance of CCPE in analyzing the datasets with dropout events, we generated three simulated datasets with different dropout rates. CCPE had a strong capability to predict cell cycle states on the data with 25.6% dropouts. When the dropout rate increased, the performance of CCPE was reduced, but still outperformed than Cyclum and CYCLOPS. Cyclone, Seurat and reCAT require preliminary gene list and cannot be applied to simulated datasets, so we did not compare with these methods on our generated datasets. To further validate the performance of CCPE, we used CCPE to analyze the datasets collected from mixed cell lines treated with a cell cycle perturbation reagent nutlin. Nutlin, a selective *MDM2* inhibitor and *MDM2* is a negative regulator of the tumor-suppressor gene *TP53*. McFarland *et al.* (29) used Seurat to identify the cell cycle phase of each cell and concluded that Nutlin elicits rapid apoptosis and cell cycle arrest in G1 phase exclusively in the *TP53* wild-type cells compared with the untreated cells. CCPE successfully caught the G1 arrest induced by nutlin in *TP53* WT cells. Differential gene expression analysis further validated the accuracy of CCPE in estimating cell cycle phases. Removing cell cycle-related genes inferred by CCPE enhances differences between two phenotypes for 416B dataset.

CCPE is an unsupervised machine learning method and does not require cell-type specific or single cell sequencing-method specific information as input. Therefore, CCPE can be applied to analyze various scRNA-seq data. In our studies, we applied simulated datasets and real datasets obtained by different single-cell sequencing methods to evaluate the performance of CCPE, such as mESCs Quartz-Seq dataset using Quartz-Seq technology, H1 hESCs scRNA-seq dataset and E-MTAB-2805 mESCs dataset using Smart-seq technology, Nutlin-treated multiple cancer cell line dataset using 10× technology and 416B cell line scRNA-seq dataset using Smart-seq2 technology. This illustrates the applicability of CCPE to different types of single cell RNA-seq data. The CCPE model is also designed based on the modeling of circular or periodic processes, therefore, it is not limited to deal with cell cycle problems, it can also do pseudotime analysis of any periodic biological processes, such as circadian rhythms, self-renewal processes, etc. In future studies, we plan to use CCPE to study mechanisms involving both linear and nonlinear components, such as cell heterogeneity combined with cell cycle modeled by nonlinear components and cell types modeled by linear components from scRNA-seq data. In addition, CCPE uses soft clustering methods instead of hard clustering assignments to obtain cell cycle discriminative information, so that to achieve smooth transitions between cell states and between different cell cycle phases. The soft clustering algorithm favors clusters with cells from multiple datasets and preserves discrete and continuous topologies, while avoiding local minima caused by excessively maximizing the representation on multiple datasets (55). The application of soft clustering in CCPE inspires the potential of CCPE to predict the cell cycle of datasets with different experimental and biological batches, which is what we plan to investigate next.

## DATA AVAILABILITY

CCPE is an available tool in the GitHub repository (https://github.com/LiuJJ0327/CCPE). The drtoolbox (56,57) was used in CCPE to initialize the embedded expression profiles $Z$ and it is available from https://lvdmaaten.github.io/drtoolbox/. The source of the datasets we used is described in Materials and Methods.

## Supplementary Material

gkab1236_Supplemental_Files
