# Supplementary material for: CCPE: cell cycle pseudotime estimation for single cell RNA-seq data
Source: Nucleic Acids Res. 2021 Dec 21;50(2):704–16. doi: 10.1093/nar/gkab1236 (PMC8789092; doi:10.1093/nar/gkab1236)
Supplement: gkab1236_Supplemental_Files [file gkab1236_supplemental_files.zip › Supplementary_Figures.pdf]

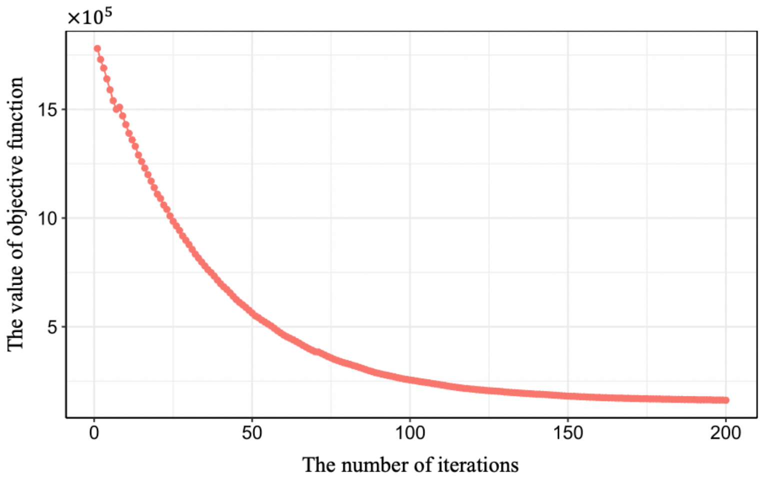

Supplementary Figure S1. The convergence analysis of the objective function in CCPE based on E-MTAB-2805 mESCs dataset.

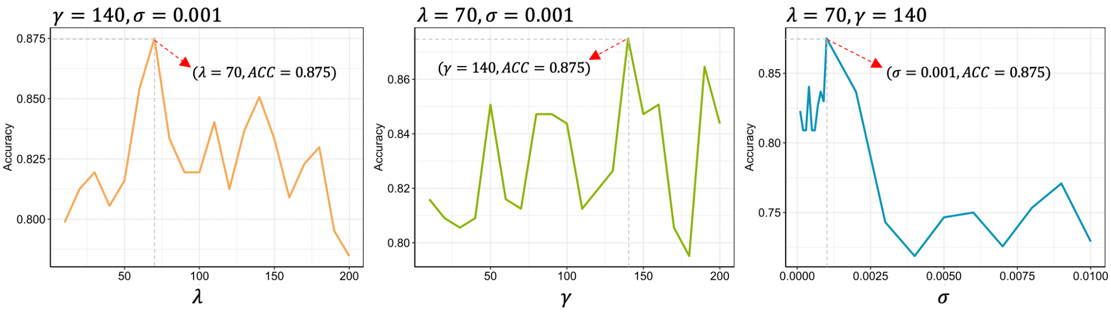

Supplementary Figure S2. The determination of weighting parameters in the objective function of CCPE based on E-MTAB-2805 mESCs dataset.

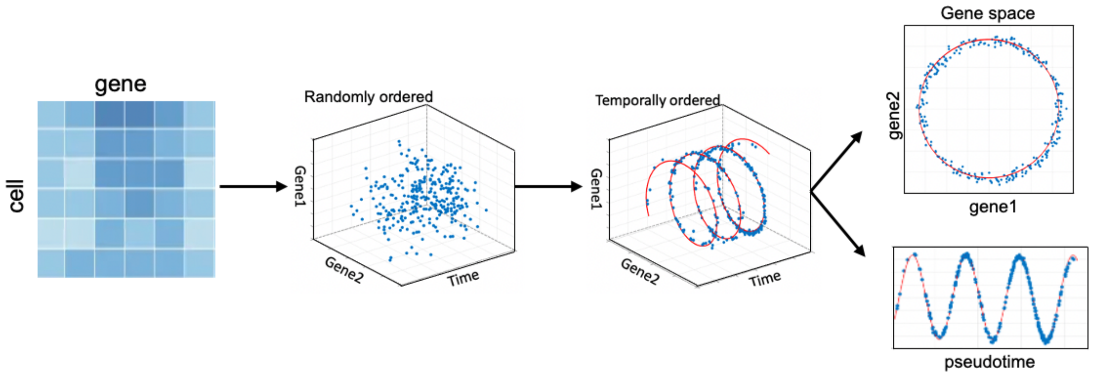

Supplementary Figure S3. Graphic description of CCPE. Randomly distributed cells were ordered by CCPE along a learned helix, with 2D-gene space capturing the cell cycle process and one dimension representing the inferred cell cycle pseudotime.

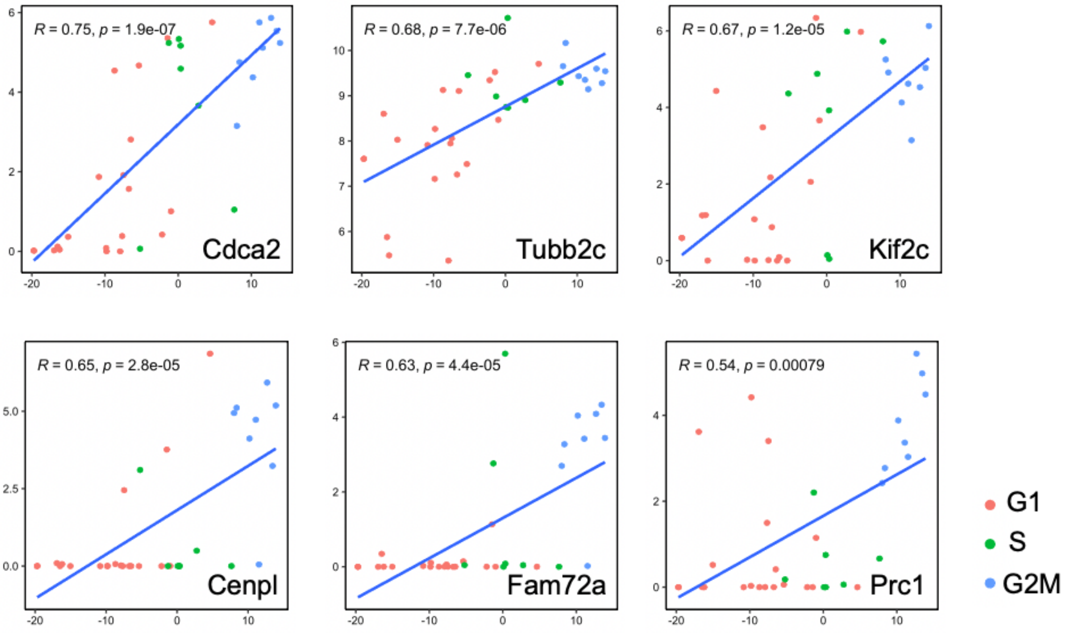

Supplementary Figure S4. Correlations of expression of cell cycle marker genes and CCPE-inferred pseudotime. The correlation coefficients and p-value are shown on the top left of each figure. Marker genes with correlation coefficients greater than 0.5 are showed.

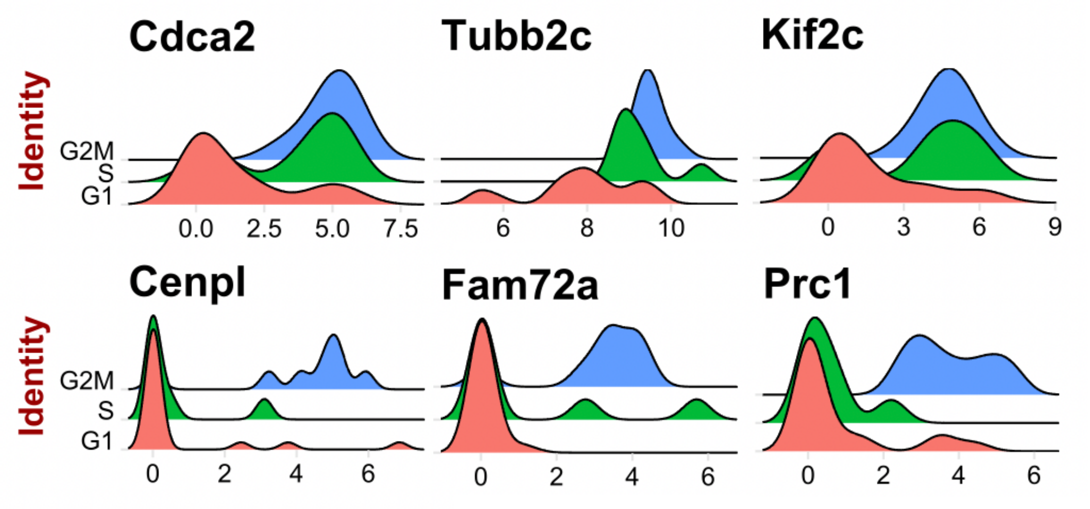

Supplementary Figure S5. Seurat plot of cell cycle marker genes with correlation over 0.5.

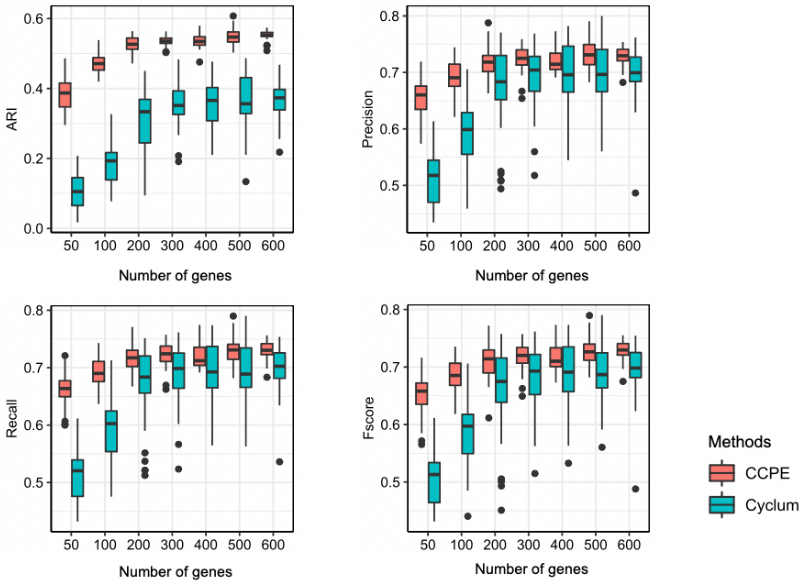

Supplementary Figure S6. Boxplots of ARI, Precision, Recall and Fscore values indicate the performance of CCPE and Cyclum on the subsampled datasets with smaller number of genes.

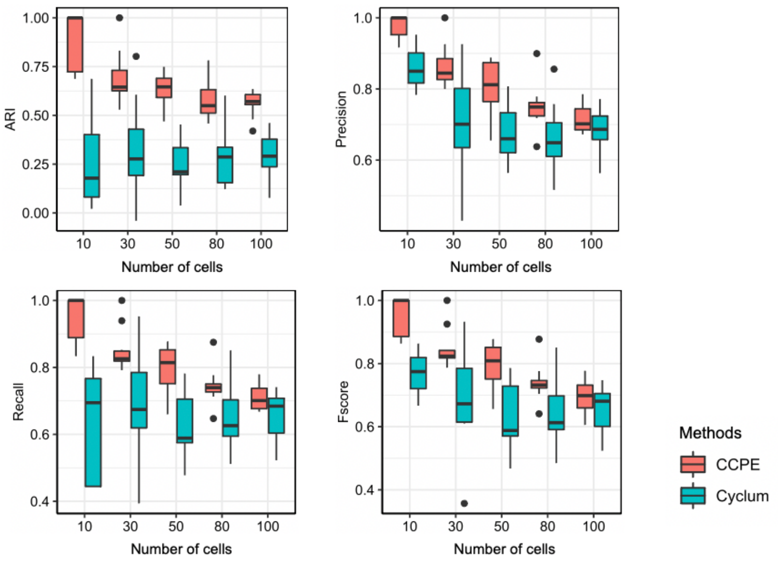

Supplementary Figure S7. Boxplots of ARI, Precision, Recall and Fscore values indicate the performance of CCPE and Cyclum on the subsampled datasets with different numbers of cells.

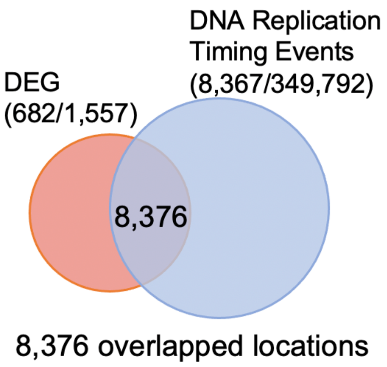

Supplementary Figure S8. Venn diagram shows the overlap of CCPE-DEGs and DNA Replication Timing Events. 682 out of 1,557 DEGs overlapped with 8,367 DNA Replication Timing Events and generate 8,376 overlapped locations.

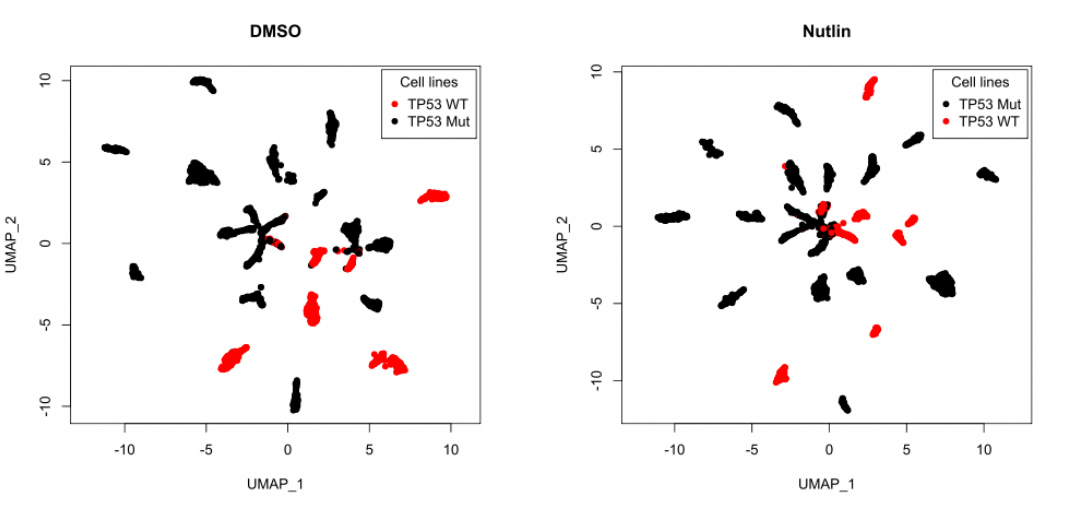

Supplementary Figure S9. UMAP plots for cancer cell lines treated with DMSO and nutlin, separately. Cells were colored by TP53 Mut and TP53 WT cell line types.

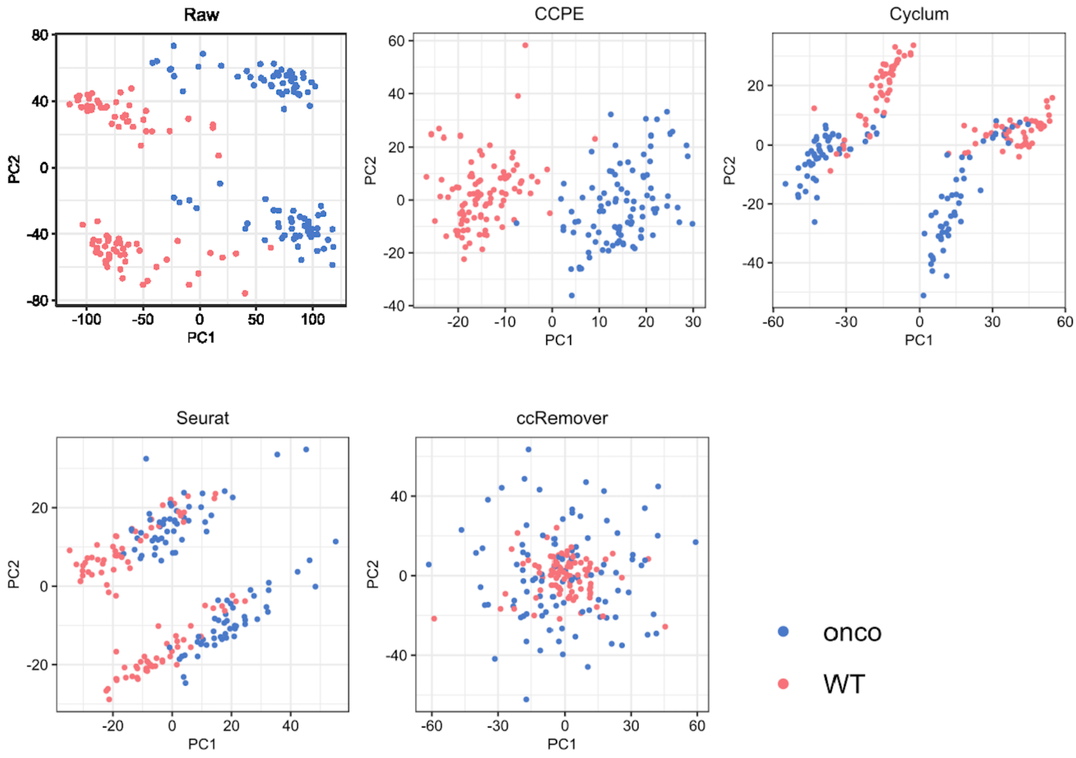

Supplementary Figure S10. Visualizations of the 416B dataset, generated before and after removing cell cycle effect using CCPE, Cyclum, Seurat and ccRemover. Each point corresponds to a cell that is colored by oncogene induction status.

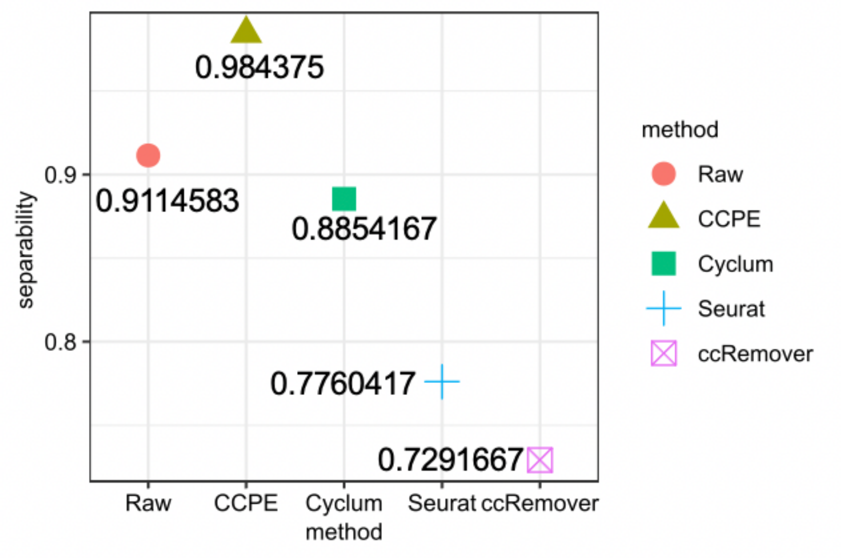

Supplementary Figure S11. The separability of phenotypes of 416B cell line scRNA-seq dataset, generated before and after removing cell cycle effect using CCPE, Cyclum, Seurat and ccRemover.
