# Supplementary material for: CCPE: cell cycle pseudotime estimation for single cell RNA-seq data
Source: Nucleic Acids Res. 2021 Dec 21;50(2):704–16. doi: 10.1093/nar/gkab1236 (PMC8789092; doi:10.1093/nar/gkab1236)
Supplement: gkab1236_Supplemental_Files [file gkab1236_supplemental_files.zip › Supplementary_Tables.pdf]

Supplementary Table S1: Datasets used in CCPE

Please see the excel file Supplementary Table S1.xlsx

Supplementary Table S2: dimension of each matrix in the objective function

matrix	dimension
X	$D \times N$
$Z, \hat{Z}, C$	$d \times N$
W	$D \times d$
Y	$d \times K$
R	$N \times K$
$\Gamma = diag(1^T R)$	$K \times K$
Q	$N \times N$
$A = \hat{Z} Q X^T$	$d \times D$
U	$d \times d$
V	$D \times D$
Σ	$d \times D$

Supplementary Table S3: ANOVA result for pseudotime in G1, S and G2/M phases

Groups	Sum	Mean	Variance
**G1**	-184.92675	-9.2463375	43.3654923
**S**	4.638156	0.66259371	15.3583825
**G2/M**	89.2422	11.155275	4.82132256

Supplementary Table S4: Values of ten times clustering metrics for mESCs Quartz-seq data using different methods

Please see the excel file Supplementary Table S4.xlsx

Supplementary Table S5: Values of ten times clustering metrics for E-MATB-2805 mESCs data using different methods

Please see the excel file Supplementary Table S5.xlsx
