# Supplementary material for: CCPE: cell cycle pseudotime estimation for single cell RNA-seq data
Source: Nucleic Acids Res. 2021 Dec 21;50(2):704–16. doi: 10.1093/nar/gkab1236 (PMC8789092; doi:10.1093/nar/gkab1236)
Supplement: gkab1236_Supplemental_Files [file gkab1236_supplemental_files.zip › Supplementary_Texts.pdf]

Supplementary Text S1: Simplification of the objective function

To make it easy to optimize the objective function in CCPE, we transform Equation (4) into the simplified object function, Equation (11) in our manuscript by fixing $\hat{Z} = C$,. Below we give the transformed process

$$L = \min_{W,Z,Y} \sum_{i=1}^{N} \|X_i - WZ_i\|^2 + \lambda \sum_{i=1}^{N} \left\|Z_i - \widehat{Z_i}\right\|^2 + \gamma\left[\sum_{k=1}^{K} \sum_{i=1}^{N_k} r_{i,k} \|Z_i - Y_k\|^2 + \sigma\Omega(R)\right]$$

$$= \min_{W,Z,Y} tr[(X - WZ)(X - WZ)^T + \lambda(Z - C)(Z - C)^T + \gamma(ZZ^T - 2R^T Z^T Y - Y\Gamma Y^T)]$$

$$= \min_{W,Z,Y} tr[XX^T - 2W^T XZ^T + (1 + \lambda + \gamma)ZZ^T - 2\lambda CZ^T + \lambda CC^T - 2\gamma R^T Z^T Y + \gamma Y\Gamma Y^T]$$

where $\Gamma = diag(1^T R)$ and R is the matrix of $r_{i,k}$. Set the first derivative of L with respect to Y to zero

$$\frac{\partial L}{\partial Y} = -2\gamma ZR + 2\gamma Y\Gamma = 0$$

$$Y = ZR\Gamma^{-1}$$

Substituting Y into L

$$L = \min_{W,Z} tr[XX^T - 2W^T XZ^T + (1 + \lambda + \gamma)ZZ^T - 2\lambda CZ^T - \gamma ZR\Gamma^{-1} R^T Z^T$$

Set the first derivative of L with respect to Z to zero

$$\frac{\partial L}{\partial Z} = -2W^T X + 2(1 + \lambda + \gamma)Z - 2\lambda C - 2\,\gamma ZR\Gamma^{-1} R^T = 0$$

$$Z = (W^T X + \lambda C)[(1 + \lambda + \gamma)I - \gamma R\Gamma^{-1} R^T]^{-1}$$

Set $Q = [(1 + \lambda + \gamma)I - \gamma R\Gamma^{-1} R^T]^{-1}$, then $Z = (W^T X + \lambda C)Q$. Substituting Z into L

$$L = \min_{W} tr(M - 2\,\lambda CQX^T W)$$

where $M = XX^T - \lambda^2 CQ^T C^T$ is independent of W and $\lambda > 0$, so our objective function is simplified into the problem

$$L = \max_{W} tr(CQX^T W) \colon W^T W = I$$

Supplementary Text S2: Prove that the inverse of matrix Q exists

We provide the simplification of the objective function based on the assumption that the inverse of matrix $[(1 + \lambda + \gamma)I - \gamma R\Gamma^{-1} R^T]$ in the formula of Q exists. According to the Woodbury matrix identity [1], we obtain the following equation

$$Q = [(1 + \lambda + \gamma)I - \gamma R\Gamma^{-1} R^T]^{-1}$$

$$= (1+\lambda+\gamma)I + \gamma(1+\lambda+\gamma)R[\Gamma - \gamma(1+\lambda+\gamma)RR^T]^{-1}(1+\lambda+\gamma)R^T$$

Now the problem becomes into the proof that the inverse of $[\Gamma - \gamma(1+\lambda+\gamma)RR^T]$ exists. Given any non-zero vector $x \in R^K$, we have the following derivations

$$x^T[\Gamma - \gamma(1+\lambda+\gamma)RR^T]x = x^T\Gamma x - \gamma(1+\lambda+\gamma)x^T RR^T x$$

$$= x^T\Gamma x + \gamma(1+\lambda+\gamma)x^T(diag(1RR^T) - RR^T)x - \gamma(1+\lambda+\gamma)x^T diag(1RR^T)x$$

$$\geq x^T\Gamma x - \gamma(1+\lambda+\gamma)x^T diag(1RR^T)x$$

$$\geq x^T\Gamma x - \gamma(1+\lambda+\gamma)x^T diag(1^T R)x$$

$$= x^T\Gamma x - \gamma(1+\lambda+\gamma)x^T\Gamma x$$

$$= [1 - \gamma(1+\lambda+\gamma)]x^T\Gamma x > 0$$

It proved that the matrix $[\Gamma - \gamma(1+\lambda+\gamma)RR^T]$ is positive definite, thus the inverse of the matrix $[\Gamma - \gamma(1+\lambda+\gamma)RR^T]$ exists. That is to say the inverse of matrix $[(1+\lambda+\gamma)I - \gamma R\Gamma^{-1}R^T]$ in the formula of Q exists.

Supplementary Text S3: The constrained quadratic problem

The simplified optimization function $L = \max_W tr(CQX^TW)\colon W^TW = I$ can be regarded as a constrained quadratic problem, $L = \min_W ||W - XQ^TC^T||^2\colon W^TW = I$, as is shown in the following

$$L = \min_W ||W - XQ^TC^T||^2$$

$$= \min_W tr(W - XQ^TC^T)(W - XQ^TC^T)^T$$

$$= \min_W tr(WW^T + XQ^TC^TCQX^T) - 2tr(CQX^TW)\colon W^TW = I$$

Which is equal to the optimization problem $L = \max_W tr(CQX^TW)\colon W^TW = I$. If the SVD of CQX^T is $U\Sigma V^T$, the optimization problem has the unique solution $W = VI_{D\times d}U^T$, which has been proved by Manton *et al.* [2].

Supplementary Text S4: Details of seven multiclass classification metrics

We define TP, TN, FP, FN as follows

	Predicted Class		
Actual Class		Class=Yes	Class= No
	Class=Yes	TP	FN
	Class=No	FP	TN

TP is the number of true positives, TN is the number of true negatives, FP is the number of false positives, and FN is the number of false negatives. Details of the clustering metrics used in our study are shown as follows

1. Rand Index (RI): the Rand index represents the frequency of occurrence of agreements over the total pairs, it can also represent as a measure of the percentage of correct decisions made by the algorithm. It can be computed using the following formula

   $$RI = \frac{TP + TN}{TP + FP + FN + TN}$$

   Properties: the Rand index has a value between 0 and 1, with 0 indicating that the two clusters do not agree on any pair of points and 1 indicating that the data clusters are exactly the same [3]. The disadvantage of the Rand index is that it cannot handle randomly assigned clusters. As the number of clusters increases, the Rand index of the randomly assigned clusters will increase, which should be close to zero. Therefore, we also considered the Adjusted Rand Index.

2. Adjusted Rand Index (ARI) [4]: the adjusted Rand Index is a modified version of the Rand Index [3, 5, 6]. The adjusted Rand index corrects for chance and will give a baseline for the randomly assigned clustering problem in the Rand Index. It is calculated as

   $$ARI = \frac{\sum_{ij}\binom{n_{ij}}{2} - [\sum_i \binom{a_i}{2} \sum_j \binom{b_j}{2}]/\binom{n}{2}}{\frac{1}{2}\left[\sum_i \binom{a_i}{2} + \sum_j \binom{b_j}{2}\right] - [\sum_i \binom{a_i}{2} \sum_j \binom{b_j}{2}]/\binom{n}{2}}$$

   Properties: the bounded range of the Adjusted Rand Index is [-1, 1]: negative values are bad (independent labels), similar clustering results have a positive ARI, 1.0 is the perfect match score. No assumption is made on the cluster structures [7].

3. Normalization Mutual Information (NMI): Normalized Mutual Information (NMI) is a normalization of the Mutual Information (MI) score to scale the results between 0 (no

mutual information) and 1 (perfect correlation). Mutual Information tells us the reduction in the entropy of class labels that we get if we know the cluster labels. It is calculated as

$$NMI = \frac{H(X) + H(Y) - H(X,Y)}{(H(X) + H(Y))/2}$$

where $H(X)/H(Y)$ is the entropy of the random variable X/Y associated with two partitions, whereas $H(X,Y)$ is the joint entropy [8].

Properties: It can be used to compare two clustering results that have different number of clusters

4. Accuracy: Accuracy in classification problems is the number of correct predictions made by the model over the ground truth. It is calculated as

$$ACC = \frac{TP + TN}{N}$$

N is the number of samples.

Properties: Accuracy is a good measure when the target variable classes in the data are nearly balanced. Accuracy is equal to Rand index in the binary classification, but they are different in multiclass classification in CCPE.

5. Precision: the precision in the binary classification is defined as the number of true positives TP over the number of true positives TP plus the number of false positives FP as the following

$$PREC = \frac{TP}{TP + FP}$$

In the classification of CPPE, there are three clusters representing G1, S and G2/M phases. We calculate the precision for each phase and get three precision values. The Macro-Precision [9] used in CCPE is calculated as

$$Macro - Precision = \frac{Precision_{G1} + Precision_{S} + Precision_{G2/M}}{3}$$

Properties: the precision is a measure of result relevancy and it is useful to measure the prediction of multiple classes.

6. Recall: the recall in the binary classification is defined as the number of true positives TP over the number of true positives TP plus the number of false negatives FP as the following

$$REC = \frac{TP}{TP + FN}$$

In the classification of CPPE, there are three clusters representing G1, S and G2/M phases. We calculate the recall for each phase and get three recall values. The Macro-Recall [9] used in CCPE is calculated as

$$Macro - Recall = \frac{Recall_{G1} + Recall_{S} + Recall_{G2/M}}{3}$$

Properties: the recall is a measure of how many truly relevant results are returned, also known as true positive rate.

7. Fscore: the Fscore in the binary classification is the harmonic mean of precision and recall and is calculated as

$$Fscore = 2 \cdot \frac{Precision \cdot Recall}{Precision \times Recall}$$

Macro-Fscore [9] used in CCPE for multiclass classification is calculated as

$$Macro - Fscore = 2 \cdot \frac{Macro - Precision \cdot Macro - Recall}{Macro - Precision \times Macro - Recall}$$

Properties: harmonic mean is kind of an average when precision and recall are equal.

Supplementary References

1. Horn, R.A. and C.R. Johnson, *Matrix Analysis.* 1985, Cambridge: Cambridge University Press.
2. Manton, J.H., *Optimization algorithms exploiting unitary constraints.* IEEE Transactions on Signal Processing, 2002. **50**(3): p. 635-650.
3. Rand, W.M., *Objective criteria for the evaluation of clustering methods.* Journal of the American Statistical association, 1971. **66**(336): p. 846-850.
4. Mao, Q., et al. *Dimensionality reduction via graph structure learning.* in *Proceedings of the 21th ACM SIGKDD International Conference on Knowledge Discovery and Data Mining.* 2015.
5. Hubert, L. and P. Arabie, *Comparing partitions.* Journal of classification, 1985. **2**(1): p. 193-218.
6. Vinh, N.X., J. Epps, and J. Bailey, *Information theoretic measures for clusterings comparison: Variants, properties, normalization and correction for chance.* The Journal of Machine Learning Research, 2010. **11**: p. 2837-2854.
7. Yeung, K.Y. and W.L. Ruzzo, *An empirical study on principal component analysis for clustering gene expression data.* Bioinformatics, 2001. **17**(9): p. 763-774.
8. Lancichinetti, A., S. Fortunato, and J. Kertész, *Detecting the overlapping and hierarchical community structure in complex networks.* New journal of physics, 2009. **11**(3): p. 033015.
9. Sokolova, M. and G. Lapalme, *A systematic analysis of performance measures for classification tasks.* Information Processing & Management, 2009. **45**(4): p. 427-437.
